# P-250. Identifying the Risks Associated with Increased Tissue Expander Infections Amongst Patients Undergoing Mastectomy with Immediate Tissue Expander Placement

**DOI:** 10.1093/ofid/ofae631.454

**Published:** 2025-01-29

**Authors:** Courtney Koplyay, Madeline Ngo, Tessy Davidson, Madhuri Sopirala

**Affiliations:** UT Southwestern Medical Center, Dallas, Texas; UT Southwestern Medical Center, Dallas, Texas; UT Southwestern Medical Center, Dallas, Texas; UT Southwestern Medical Center and Parkland Health, Dallas, Texas

## Abstract

**Background:**

Tissue expander (TE) infections are increasingly common in patients undergoing mastectomy with immediate TE placement. Post-mastectomy TE infections prolong recovery time, delay second-stage reconstruction, and confer a significant socioeconomic burden on the patient. In this study, we aim to identify modifiable risk factors associated with TE infections following TE manipulation and assess the infection risk associated with different TE implants (Mentor™ vs. Sientra™).

Univariate Analysis of Modifiable Risk Factors

**Figure:**
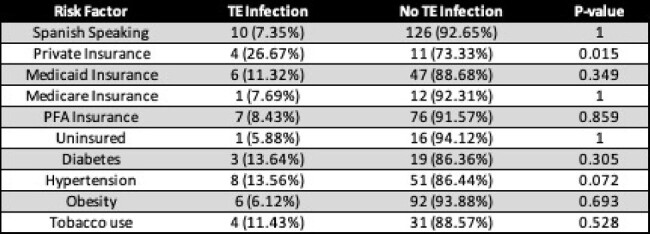

Univariate analysis of modifiable risk factors associated with the development of tissue expander (TE) infection.

**Methods:**

We identified several lapses in sterile technique during TE manipulation procedures performed at the plastic surgery clinic of a large safety net hospital. We conducted a retrospective review of all adult patients who underwent mastectomy with immediate TE placement and expansion from 2019-2022, collecting demographic, co-morbidity, and infection surveillance data. Cases were defined as patients who developed an infection after a clinic-based TE manipulation procedure and controls as patients who did not develop an infection after a clinic-cased TE manipulation procedure.

Multivariate Logistic Regression of Modifiable Risk Factors

**Figure:**
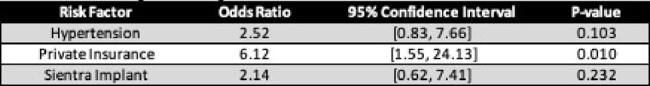

Multivariate logistic regression analysis of modifiable risk factors associated with tissue expander (TE) infection.

**Results:**

Of 292 cases, 16 developed a TE infection after TE manipulation. The total post-manipulation TE infection rate is 6.1 per 100 procedures. The type of implant did not influence infection rates on multivariate analysis (p=0.23). Patients who developed an infection after TE manipulation were more likely to have private insurance than those who did not develop an infection (p<0.01). There was no significant difference in the presence of hypertension, diabetes, obesity, and tobacco use in patients who developed a TE infection compared to those who didn’t develop a TE infection.

**Conclusion:**

There were no identifiable modifiable risk factors associated with increased rates of TE infections following TE manipulation. We found that private insurance was not protective against the development of a TE infection. Suboptimal sterile access to TEs may independently contribute to post-mastectomy TE infections. Implementation of a new sterile technique has yet to confer a significant decrease in TE infection rates. We will continue refining our standardized sterile protocol as we monitor TE infection rates.

**Disclosures:**

**All Authors**: No reported disclosures

